# Supplementary figures and images for: Profiling transcription factor activity dynamics using intronic reads in time-series transcriptome data
Source: PLoS Comput Biol. 2022 Jan 10;18(1):e1009762. doi: 10.1371/journal.pcbi.1009762 (PMC8782462; doi:10.1371/journal.pcbi.1009762)

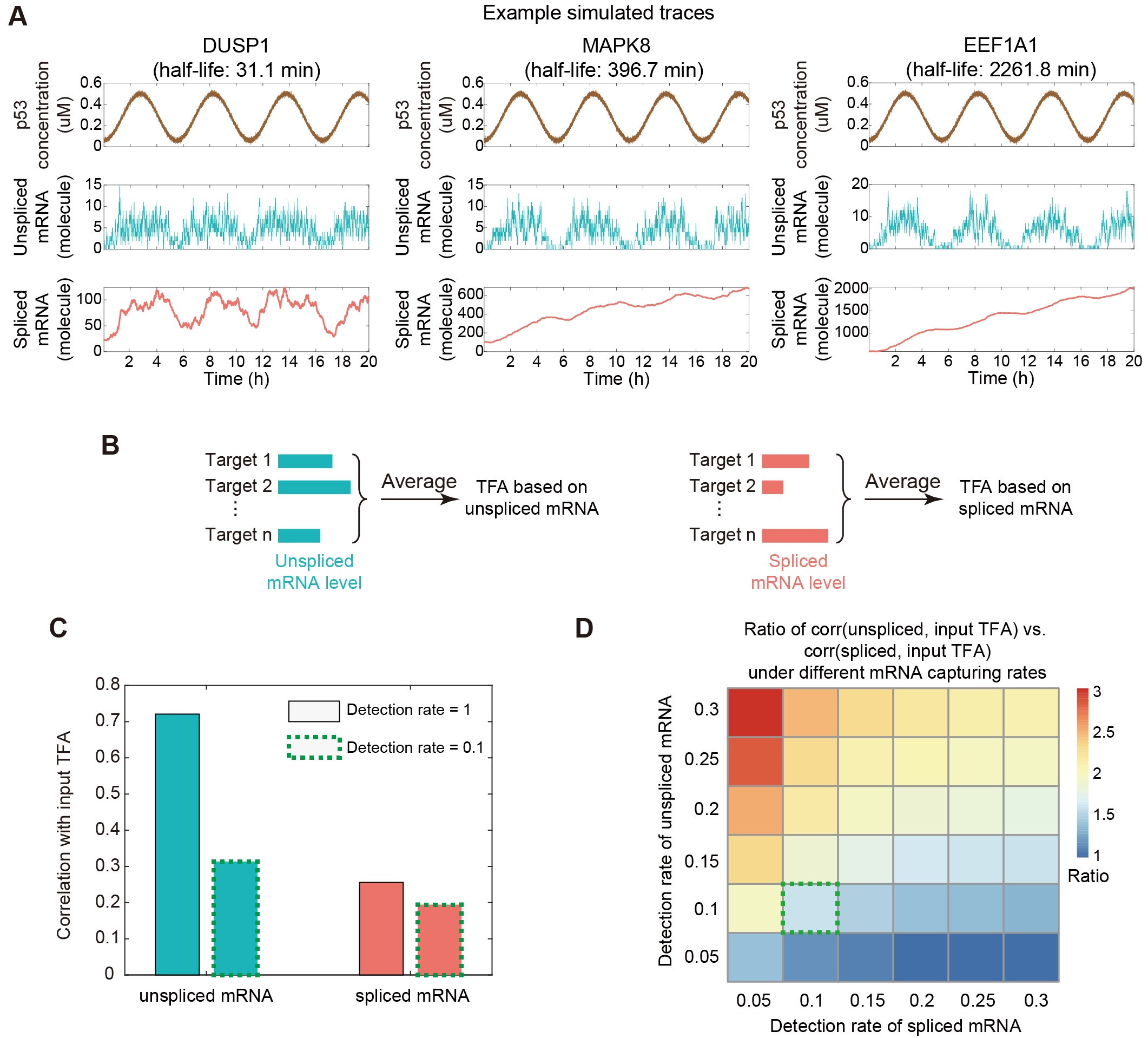

Supplement: S1 Fig — (A) Representative simulated traces of p53 dynamics, unspliced mRNA dynamics and spliced mRNA dynamics of three example genes. The spliced mRNA dynamics of the three genes are different due to their different mRNA half-lives. (B) The pipeline for calculating TFA using target expression levels from the simulation. The average expression level of target genes in the regulon of a TF is used as the estimate of the TF’s activity. Either unspliced or spliced mRNA expression level can be used in the calculation. (C-D) The effect of detection rate (i.e., mRNA capture rate) on the correlation of gene expression level with input TFA. A p53 target gene was simulated at varying detection rates (such as 10% in C, see Materials and Methods), and the correlation between unspliced or spliced mRNA and input TFA was shown (C). The ratio between the two correlations were calculated for all simulated pairs of detection rates (D). (TIF) [file pcbi.1009762.s001.tif]

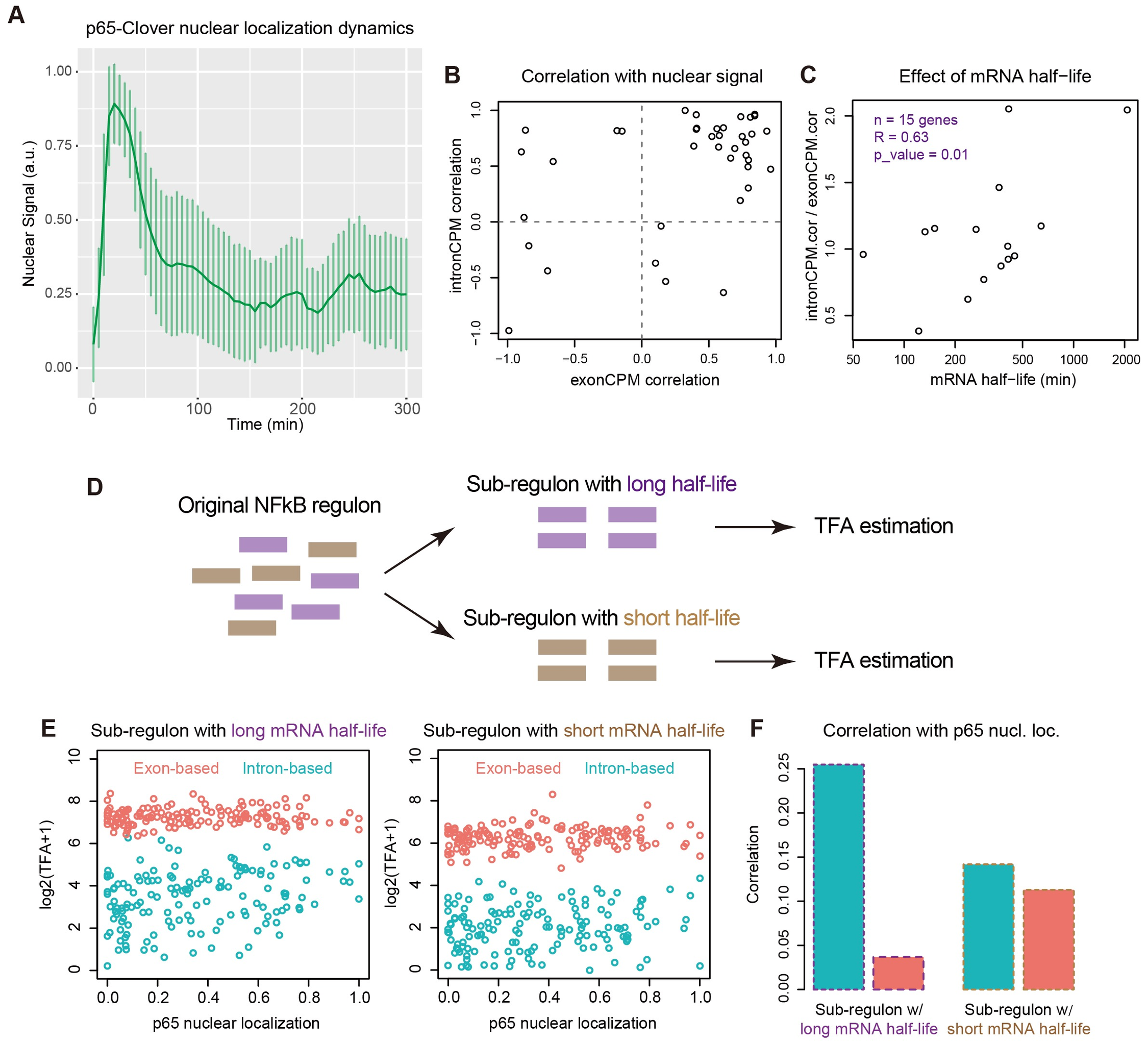

Supplement: S2 Fig — (A) Population-averaged NF-κB nuclear localization dynamics of the data from Lane et al. n = 637 cells for t = 0–75 min, n = 492 cells for t = 80–150 min, and n = 124 cells for t = 155–300 min. Error bar represents the standard deviation. (B) Analysis of individual NF-κB target genes. We focused on high-expressing genes (exonCPM >1 & intronCPM >1). In 39 high-expressing target genes, 26 genes showed positive correlation for both intronCPM and exonCPM. See Materials and Methods for details. (C) The effect of mRNA half-life on the relative performance of intron-based method versus exon-based method. For the 26 genes with double-positive correlation in (B), 15 of them have mRNA half-life data. The ratio between the intron-based correlation and exon-based correlation was plotted against mRNA half-life. Pearson correlation (R) and the associated p-value were indicated. (D) Schematic showing the division of NF-κB regulon into two sub-regulons. (E-F) Comparison of using the two sub-regulons for TFA estimations. Scatter plots showing TFAs estimated using the two sub-regulations versus NF-κB (p65) nuclear localization level in individual cells at 75 min post LPS stimulation (E). The correlations between estimated TFAs and TF nuclear localization were shown for both sub-regulons (F). (TIF) [file pcbi.1009762.s002.tif]

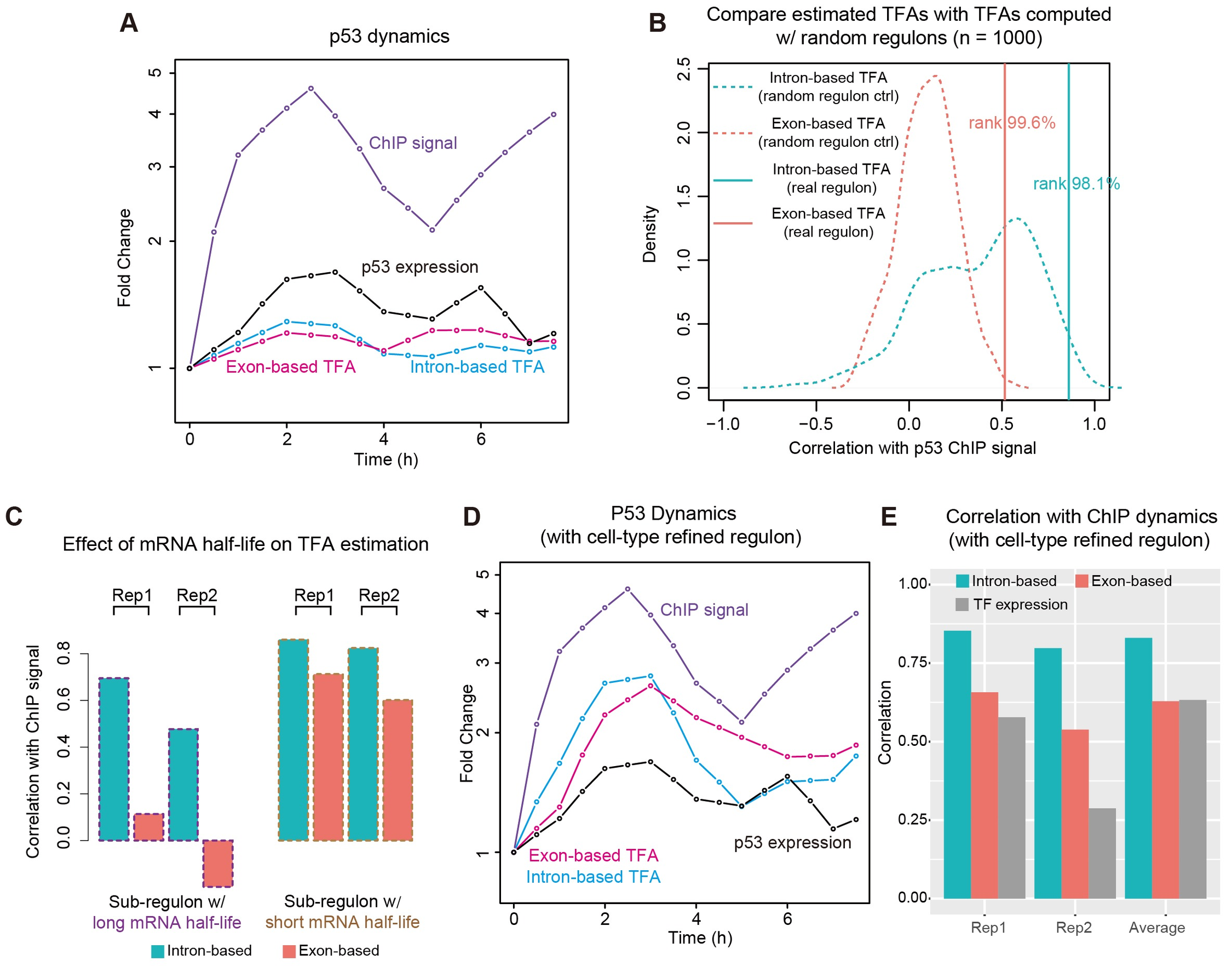

Supplement: S3 Fig — (A) Dynamics of p53 DNA binding activity, intron-based TFA, exon-based TFA and p53 expression (averages of two replicates). These dynamics were analyzed from the data of Hafner et al. (B) The comparison between estimated TFAs and control TFAs. The distribution of the control TFAs (dotted lines) were calculated from 1000 random regulons sampled from non-p53 target genes (see Materials and Methods). The vertical lines indicate the estimated TFAs using the actual p53 regulon. (C) Comparison of using the two sub-regulons containing p53 target genes with either long or short mRNA half-lives for TFA estimations. The sub-regulons were generated as in S2D Fig. See Materials and Methods for details. (D-E) p53 activity estimations using the refined p53 regulon. The cell-type-refined p53 regulon was obtained as described in the Materials and Methods. This refined regulon was then used to generate plots analogous to panel A (D) and Fig 2E (E) (TIF) [file pcbi.1009762.s003.tif]

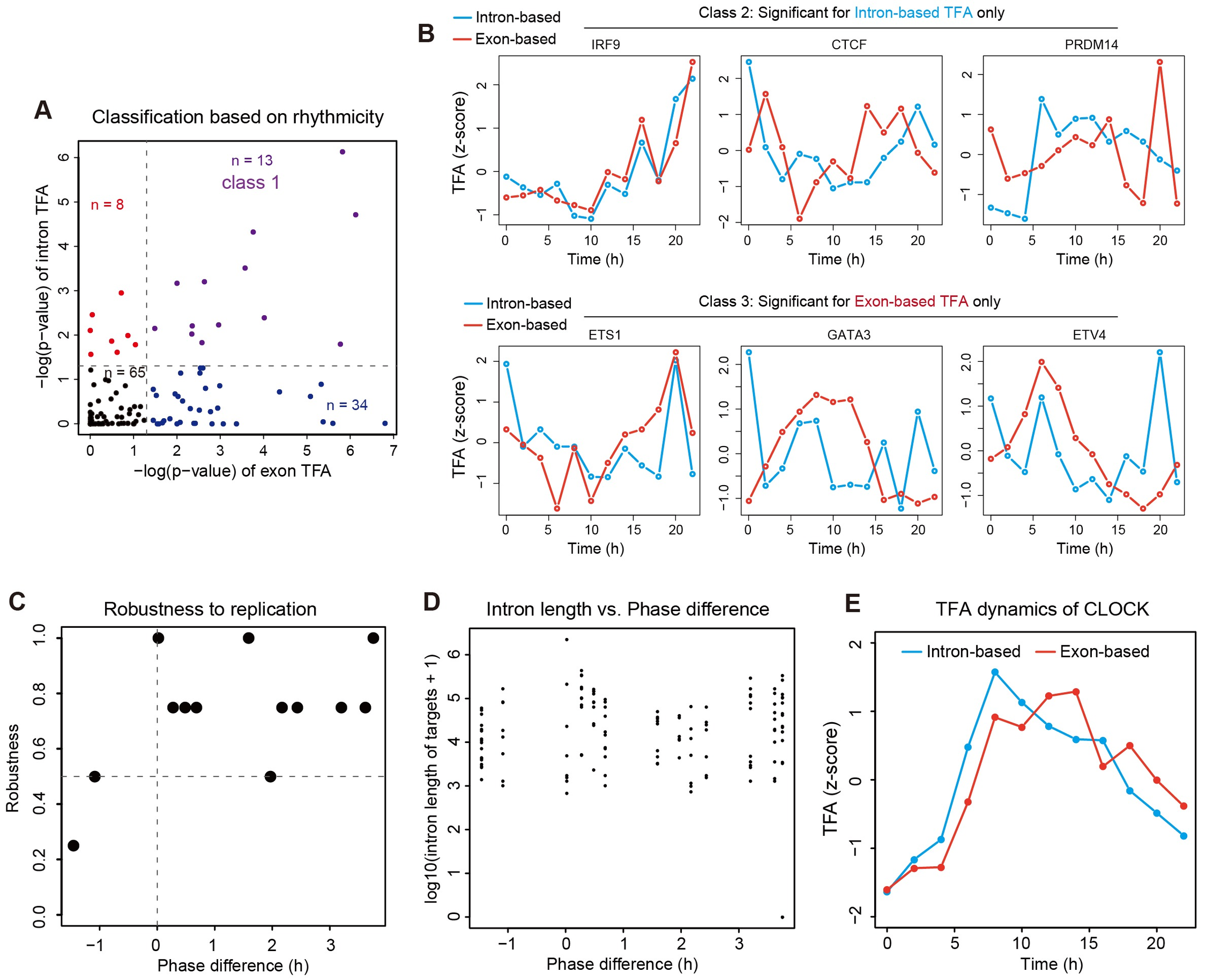

Supplement: S4 Fig — (A) Classification of TFs based on the periodicity of TFA. P-values of the periodicity were calculated using intron-based and exon-based TFAs. Black points represent non-circadian TFs by both TFAs. Red points represent circadian TFs identified by intron-based TFA. Blue points represent circadian TFs identified by exon-based TFA. Purple points represent circadian TFs identified by both methods, which were used for the analysis in Fig 3. (B) Example TFA dynamics of TFs from class 2 and class 3. Class 2 TFs were taken from the red points in panel A, while class 3 TFs were taken from the blue points. (C) Robustness of circadian TFs to experimental replication. By down-sampling of 4 replicates, the robustness of each circadian TF was defined as the fraction of attempts that the TF was identified as circadian TF. (D) Target intron lengths of TFs with different phase differences. (E) Intron-based versus exon-based TFA dynamics of CLOCK protein. Note that the regulon of CLOCK is only available when loosening the confidence threshold of the regulatory links to the third grade (i.e., score C) in the DoRothEA database. (TIF) [file pcbi.1009762.s004.tif]

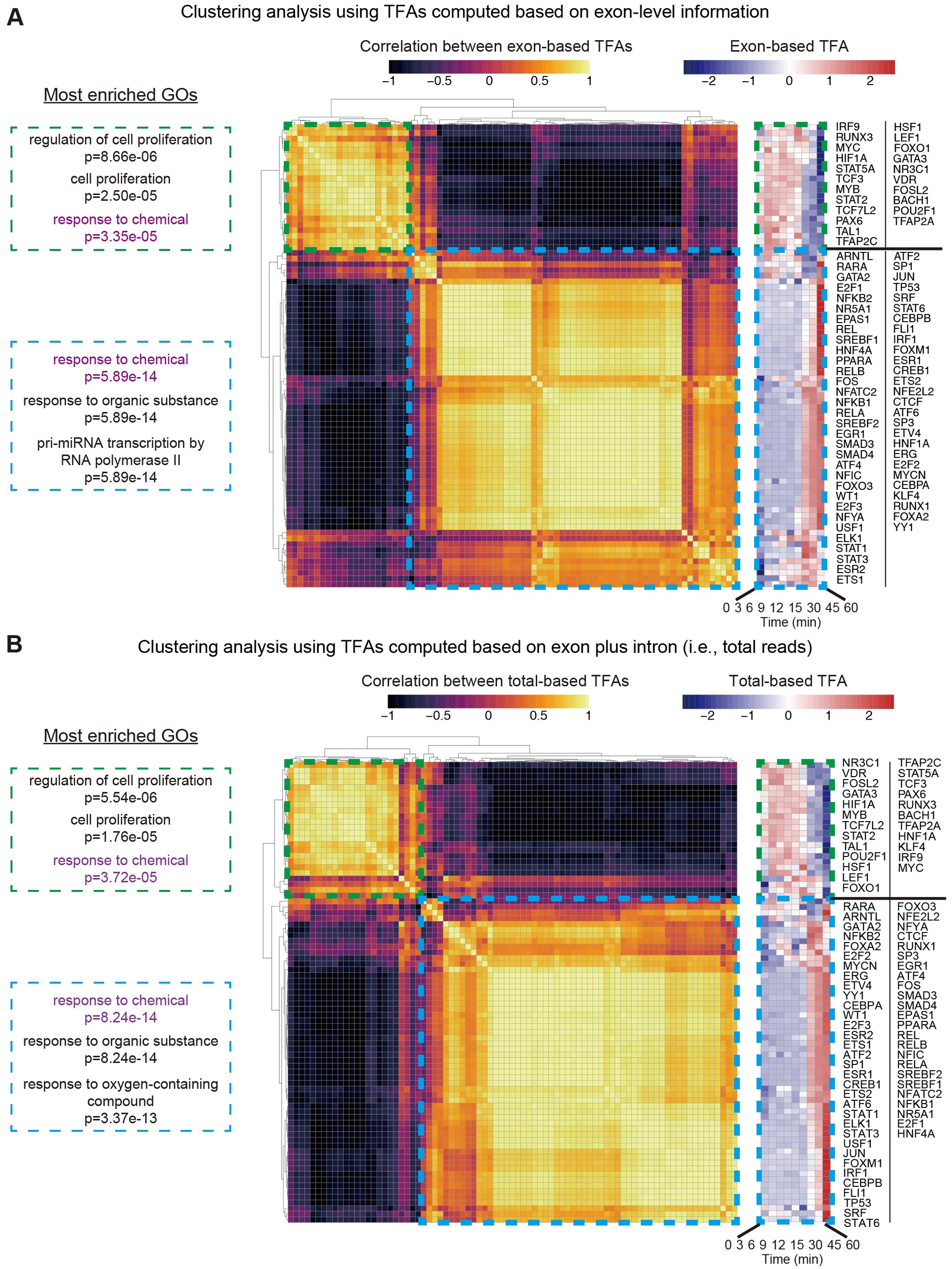

Supplement: S5 Fig — Hierarchical clustering analyses for exon-based TFA (A) and total reads-based TFA (B). These analyses are analogous to Fig 4B. Two TF modules in each panel are indicated by dashed boxes. Three most enriched GO biological process terms for each module are shown. The GO highlighted in purple indicates a shared GO between two modules in each panel. Exon-based TFA dynamics and total reads-based (exon plus intron) TFA dynamics after z-score normalization of each TF are shown on the right. Note that TF symbols are ordered from top to bottom, then left to right. (TIF) [file pcbi.1009762.s005.tif]

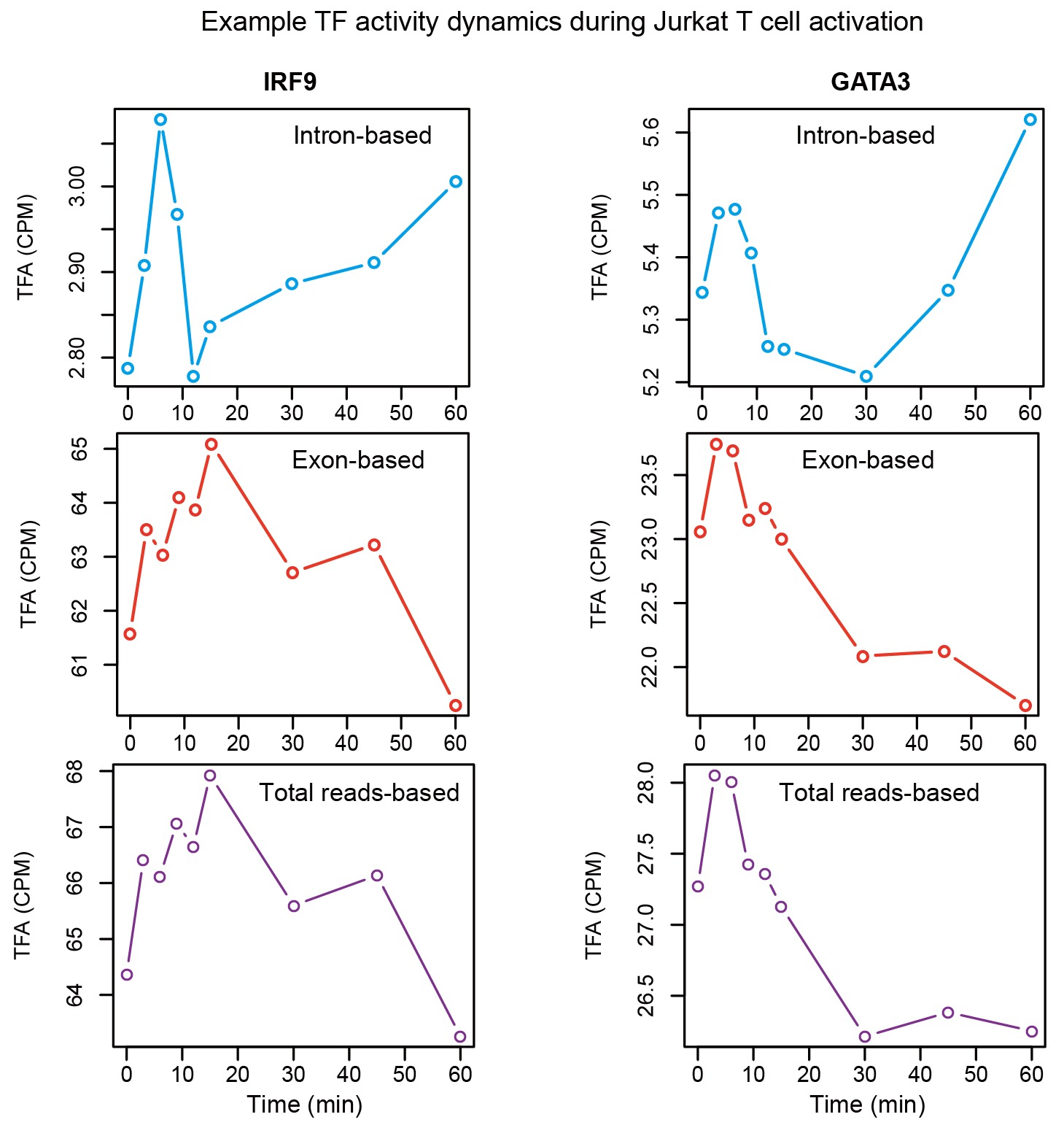

Supplement: S6 Fig — For each TF, the activity dynamics estimated by three different methods were calculated using the time-series transcriptome data (Fig 4). These two TFs were chosen as examples that the intron-based method can yield drastically different activity dynamics as compared to the other two methods. Note that the exon-based and total reads-based methods generally produce very similar activity dynamics. (TIF) [file pcbi.1009762.s006.tif]
