# Supplementary material for: Profiling transcription factor activity dynamics using intronic reads in time-series transcriptome data
Source: PLoS Comput Biol. 2022 Jan 10;18(1):e1009762. doi: 10.1371/journal.pcbi.1009762 (PMC8782462; doi:10.1371/journal.pcbi.1009762)
Supplement: Attachment — Submitted filename: 2021-11-16-response_letter.pdf [file pcbi.1009762.s009.pdf]

Dear Reviewers,

We greatly appreciate your time and the constructive comments and suggestions, which have truly helped us to improve the manuscript. In response to your comments and suggestions, we have performed a series of new simulations and analyses, and have obtained additional evidences supporting the validity and usefulness of our method. We believe that, with these new results, our work has demonstrated that intron-based transcription factor (TF) activity estimation can provide new insights into the dynamics of TFs.

In the revised manuscript, we have incorporated these new results, and have largely improved the text and figures to comprehensively address all your comments. These are described in the point-by-point reply. Here we first summarize the major changes in the revision:

- We have performed new simulations and new analyses of experimental data showing that data sparsity, mRNA stability, and regulon choice are three key factors affecting the performance of TF activity estimation. Importantly, we have also shown that the performance can be improved by refining the regulon.
- We have performed thorough comparisons between intron-based method, exon-based method, and total reads-based (i.e., intron plus exon) method. These results suggest that intron-based method can capture instantaneous TF activities better than the other two methods.
- We have further clarified the biological insights provided by intron-based method, and have included additional discussions on the applicability and limitation of the method.

Below please find a summary of changes to the figures:

1. Figure 1: Added labels to clarify that results were from simulations.
2. Figure 2: Added a sub-panel in panel B to show the estimated TFAs using total reads-based method.
3. Figure 4: GOs and adjusted p-values were updated after correcting an error in the original GO analysis code.
4. Figure S1: Added label to indicate that results were from simulations; Added panels C and D to include new simulation results on the effect of data sparsity (i.e., by simulating with low mRNA capture rates).
5. Figure S2: The panels in the original Figure S2 were splitted into new Figure S2 and Figure S3; New Figure S2 contains new plots for additional characterizations of the NF-kB system.
6. Figure S3: The original Figure S3 now becomes Figure S4; New Figure S3 now contains new plots for additional characterizations of the p53 system.
7. Figure S4: Originally Figure S3; Figure S4 now contains additional panel showing example dynamics of class 2 and class 3 TFs.
8. Figure S5: Originally Figure S4; Figure S5 now contains additional panel showing clustering analysis with total reads-based TFA method. Note that the clustering analysis of the exon-based method has also been updated as in Figure 4.
9. Figure S6: A new supplement figure to show example TF dynamics during T cell activation.

Reviewer #1: In this manuscript, Wu et al. systematically compared the intronic reads and exonic reads for TF activity inference analysis in both simulated and real time-series RNA-seq data. Sufficient evidence is provided to support their main conclusion that the intron-level information better recapitulates TF activities. They further applied this strategy to identify main TF modules regulating T cell activation in a newly generated dataset. I am generally satisfied with the main analysis and the quality of the new dataset included in the manuscript. Following are comments that should be fixed before the publication of this manuscript.

We thank the referee for the very helpful comments and suggestions. Based on these comments and suggestions, we have performed additional simulations and analyses to address the issues regarding data sparsity, alternative gene quantification method, among others. We have also added new text to clarify the limitations and the biological insights. In the following we provide a detailed point-by-point response to the comments.

1. The intronic-information significantly outperforms the spliced mRNA in the simulated data (fig. 1), but the different is not so dramatic in the real dataset. The specific reasons should be discussed and clarified in the manuscript.

Thank you for raising the issue regarding the not-so-dramatic improvement of intron-based TFA when applied to the experimental data, which should certainly be clarified. Indeed, intron-based TFA largely outperforms exon-based TFA in simulations, while the improvement is not as large when using experimental data. The specific reasons for such a difference are at least two-fold:

- First, in typical RNA-seq datasets (either bulk or single-cell) the fraction of reads that is mapped to introns is smaller than the fraction mapped to exons (e.g., ~30% vs. ~70%). Consequently, the intronic read counts for specific genes are generally smaller than exonic read counts, as pointed out in the referee's second comment. Thus, intronic read counts are more susceptible to noises, especially for single-cell RNA-seq, and can lead to inaccuracies in the estimated TFA. Additional analyses on this issue are included in the response to the second comment.
- The second potential reason relates to the limitation in the accuracy of the regulon. As we described in the manuscript, the regulons were taken from the DoRothEA database. Although this database was carefully curated and multi-evidence-based, it is a potential source of error, as there are likely false positive targets that would reduce the accuracy of TFA estimation and contribute to the not-so-dramatic improvement of intron-based TFA. In the response to referee 3's second comment, we have shown and discussed how regulon refinement could improve TFA estimation in detail.

We have now extensively modified the text to address this concern by 1) pointing out and explaining the difference between simulated and experimental results, and 2) discussed the limitations on our method due to data sparsity and regulon choice (**Line 198-206, Line 334-337, Line 486-488,** and **Line 503-506** in the main text).

2. One potential limitation of this strategy is that the intronic signal is only a small subset of the gene expression information. How does the data sparsity of the intronic information affect the analysis? Does the same strategy works for single cell RNA-seq analysis?

We agree that one limitation of intron-based TFA is the relatively lower intron read counts compared to the exon read counts (as aforementioned), and this limitation should indeed be analyzed and clarified. More specifically,

- To address how the relatively low intronic counts can potentially affect the analysis, we carried out a series of simulations to illustrate the effect of low RNA capturing rate on TFA estimations. More specifically, we carried out simulations on the p53 system used in Figure 1, as these new simulations could be directly compared with previously simulated results shown in this figure. These new simulations aimed to evaluate how intron and exon read counts affect TFA estimation. To do so, we simulated single-cell trajectories as in Figure 1 (and Fig. S1), and mimicked the low RNA capture rate in real experiments by sub-sampling the counts at pre-defined rates (i.e., from 5% to 30% for spliced or unspliced mRNAs). These sub-sampled counts were then used for TFA estimation, and the correlations between estimated TFAs and input TFAs were computed (see Methods for more details).
- By comparing between intron-based and exon-based methods from these new results, we found that: 1) the drop in capturing rate (corresponding to lower intron or exon read counts) indeed leads to the decreased correlation with input TFA for both methods; 2) intron-based method is more sensitive to the drop in capturing rate; and 3) in general intron-based method still outperforms exon-based method (see **Fig. S1C-D**).
- These findings indicate that in RNA-seq experiments where intron capture rate is lower than exon capture rate, it is indeed likely that intron-based method may display not-so-dramatic improvement compared to exon-based method. We have now included these analyses and clarifications in the text (**Line 198-206, Line 486-488**).

For the second question regarding the applicability to scRNA-seq data, the answer is yes, as we have already demonstrated the usage of intron-based TFA for single-cell sequencing data of the NFkB system (Fig. 2A-C). In these results, we found that intron-based TFA yielded a higher correlation with the nuclear localization of NFkB, and can better capture the rise-and-fall activation dynamics of NFkB compared to exon-based TFA. To improve clarity, we have now included further discussions on the applicability of our method in the discussion section of the text (**Line 502-505**).

3. For comparison analysis, it would be helpful to also include normal gene quantification (exon + intron) as this is normally used in conventional TF inference analysis.

We thank the referee for suggesting further comparison analysis. We have carried out the suggested analysis using gene quantification by total read counts mapped to both exons and introns. By doing so, we found that when using this alternative gene quantification, the estimated TFAs are very comparable to exon-based TFAs (see **Fig. 2B, Fig. S5B, Fig. S6**), as most read counts in this

quantification are from exon reads. Based on this result, we think that intron-based TFA generally outperforms exon-based TFA as well as total reads-based TFA. In the revised manuscript, these comparison analyses have been clearly presented in the three figures cited above, which were referred to in the text **Line 240-242, Line 421-424, and Line 435-437**.

We would like to further clarify the rationale for using exon-based quantification in our original comparison, instead of using total reads-based quantification. We surveyed several papers using gene expression to estimate transcription activity (such as the SCENIC paper, ref. 22), and found that most datasets were mapped to exonic regions (instead of mapped to intronic and exonic regions). And as we showed above, total reads-based quantification is largely comparable to exon-based quantification.

4. Since the full gene expression or exonic reads can be used to infer the TF modules involved in T cell activation, what is the unique biology revealed by the intronic-level analysis? This should be clarified in the manuscript.

We thank the referee for pointing out the issue regarding biological insights. We would like to address this issue by clarifying the existing findings and performing additional analyses to yield further insights. More specifically,

- In the original manuscript, we had already presented the comparison between intron-based TFA and exon-based TFA for TF modulation identification in the T cell dataset (please refer to Fig. 4 vs. Fig. S5A). In that comparison, we found that intron-based method (Figure 4) uncovered two functionally opposing modules (i.e., one module enriched for homeostatic functions while the other module enriched for stress response functions) during T cell activation that change their activities in opposite ways, while exon-based method (Fig. S5A) yielded two overlapping functional modules (i.e., both enriched with related functions). We think these results partially address the concern raised, i.e., intron-based method can indeed reveal new biological insights compared to exon-based method.
- To further address this issue, we performed analogous analysis using intron-plus-exon gene quantification (**Fig. S5B**). We found that similar to the analysis using exon-based method, this alternative gene quantification yielded two overlapping functional modules, providing further support for our finding that intron-based method can yield unique biological insights.
- We further showed that compared to exon-based and total reads-based methods, the activation of example immune-related TFs are much more dynamic when using intron-based method (**Fig. S6**).

The clarifications and the new results have been included in the revised manuscript (**Line 421-439**), which further support the conclusion that intron-based TFA can yield biologically important knowledge that cannot be obtained by using exon-based (or intron-plus-exon-based) method.

Reviewer #2: In this article, Lin and colleagues report a method to more accurately infer transcription factor activity (TFA) from RNA-seq data, where they use the intronic reads (as opposed to exonic reads) of transcription factor target genes (regulons) as measure of transcription factor activity. They show that TFA calculated using intronic reads correlate more strongly with transcription factor activity from simulated data (Fig. 1), single-cell nuclear TF translocation imaging measurements (Fig. 2A-C), TF binding data for both p53 (Fig. 2D) and for the circadian TFs ARNTL and CLOCK (FIg. 3 and Fig. S3D). Finally, they show that TFA calculated from intronic reads show partitioning into two distinct modules upon T cell activation (Fig. 4).

I found this work manuscript to be clearly written, and the for the most part, the analysis was clearly described and soundly executed.

We thank the referee for the very helpful comments and suggestions. Based on these comments and suggestions, we have performed additional simulations and analyses to address the issues regarding additional data presentation and analyses, biological insights, among others. In the following we provide a detailed point-by-point response to the comments.

However, I found the improvements in TFA estimation using intron reads to be only marginally better compared to those obtained using the exon reads (e.g. slightly better correlations with TF binding data), and was not convinced this method was a substantial improvement that could yield new insights when applied to existing transcriptome data, which was touted as a strength of this approach.

We agree that the improvement of intron-based method over exon-based method in real datasets is not as large as in the simulated data. We have discussed the potential sources of limitation in the response to the first two comments by referee one. Despite the limitation, we have clearly demonstrated several useful applications of intron-based TF activity, and have revealed new biological insights (which was not possible when using exon-based method). More specifically,

- Intron-based method has allowed us to examine TF activity dynamics at a higher accuracy compared to exon-based method, as demonstrated for the two mammalian stress response processes (NFkB and p53). For example, intron-based TF activity captured the adaptive response of NFkB activation, while exon-based method failed to do so (Fig. 2B), which we feel is rather convincing.
- Compared to exon-based method, intron-based method more accurately captured the circadian phasing of several key TFs in the mouse liver dataset, which could become a valuable tool for analyzing the regulatory dynamics during circadian rhythm. The result also yielded new TF candidates that are potentially regulated by the circadian rhythm, which could help guide future experiments.
- When comparing intron-based TFA and exon-based TFA for TF module identification in the T cell dataset (please refer to Fig. 4B vs. Fig. S5A), we found that intron-based method (Fig. 4B) uncovered two opposing functional modules during T cell activation (i.e., one module enriched for homeostatic functions while the other module enriched for stress

response functions), while exon-based method (Fig. S5A) yielded two overlapping functional modules. This result further showed that intron-based method can indeed reveal new biological insights compared to exon-based method.

Overall, we believe that our new method has the potential to uncover new biological insights in other datasets, and would become a valuable tool for the community. Furthermore, when more reliable regulons become available, the accuracy of this tool will be further improved, which we have demonstrated in detail in response to referee 3's second comment.

Analysis of TF activity from single-cell RNA-seq in T cell activation (Fig. 4) yielded two transcription factor modules, which appears to be a new insight; however, as analysis of exon data was not shown, it is not clear whether analysis of exon reads alone, using published approaches, would have been sufficient to unveil these two TF modules.

We would like to clarify that, in Fig. S4 of the original manuscript (now becomes **Fig. S5A**), we had already performed the suggested analysis. For additional details, please refer to the following point-by-point response (as well as the response to the third comment by referee one).

Comments:
- Fig. 1: It needs to be mentioned in the figure and also the legend that these p53 TF activities and correlations are obtained from simulation data; otherwise, these results can easily be mistook as those generated from experimental data.

We thank the referee for raising this issue. We have now clearly labeled the results in Fig. 1 as "Simulation results". We have also modified the title of the figure as "**Simulation-based** comparison".

- Fig. 2: It was a really nice idea for the authors to use the data from Lane et al. for Fig. 2A-C, as this data set uniquely measures NFkB localization and target gene expression in the same single cell. I feel that the authors should make use of this data by showing intron transcript levels and nuclear localization dynamics for single cells, and at the level of individual genes. It would also be nice to show here (and elsewhere) that analyzing intronic reads better recapitulates NkB activity on long half-life transcripts versus on short half-life transcripts.

We thank the referee for suggesting additional analyses that could further support our conclusion. We have performed several analyses, which showed that indeed half-life of transcripts is a key determinant for the improvement of intron-based method over exon-based method. More specifically,

- Per the referee's suggestion, we have provided the correlation between intronic or exonic expression levels and NFkB nuclear localization at the level of individual genes. Two

correlation values of each gene were plotted in a scatter plot (**Fig. S2B**). Because of the stochasticity of transcription, individual genes' expression cannot faithfully capture TF nuclear localization level, and thus our method averages the expression across the entire regulon to alleviate transcriptional noise.

- To investigate how transcript half-life affects the analysis of the NFkB dataset, we performed two separate analyses. First, we examined how individual genes' (experimental) mRNA stabilities would affect the relative performance of using intron level versus exon level to report NF-κB nuclear localization at 75 min post LPS simulation (see Methods). Reassuringly, we found that at the individual gene level, mRNA half-life significantly correlates with the relative performance of intron versus exon (**Fig. S2B-C**). We next divided the regulon into two sub-regulons with either low or high mRNA half-life, and computed repeated the analysis as in Fig. 2B-C. We found that, for the sub-regulon with long mRNA half-life, intron-based TFA largely outperforms exon-based method (**Fig. S2E-F**). In contrast, for the sub-regulon with short mRNA half-life, the difference between intron-based and exon-based methods is not as large. These results are consistent with our simulation results, and agree with the scenario described by the referee.
- We further investigate this issue by performing analogous analyses on the p53 dataset. The results concord with the findings with NFkB dataset. More specifically, for the p53 sub-regulon with long mRNA half-life, we found that intron-based TFA largely outperforms exon-based method (**Fig. S3C**). And the improvement is not as large for the other p53 sub-regulon.

Together, we believe these results have convincingly showed that intron-based TFA can indeed more accurately capture instantaneous TF activities compared to exon-based method, especially for target genes with long transcript half-lives, which have greatly strengthened our conclusion. The text has been modified to include these results (**Line 270-283**, **Line 312-318**)

- Fig. 3 and S3. Why are some genes predicted to be oscillatory when analyzing introns or exon (Class 2 and 3)? The authors point out this category, but do not elaborate; it would be interesting to know why.

We thank the referee for pointing this perplexing phenomenon that some TFs are identified to be oscillatory by only intron-based or exon-based method, which indeed should be elaborated and clarified. We carried out additional analyses to provide insights into this phenomenon. More specifically,

- We plotted the dynamics of example TFs from the two classes of TFs that were identified to be oscillatory by only one method (**Fig. S4B**).
- We first examined the dynamics class 2 TFs showing oscillatory exon-based TFA but not intron-based TFA. We found that for these TFs, it is likely that because intron-based TFA displays dynamic patterns at the sub-circadian-cycle timescale (**Fig. S4B** top), thus the algorithm failed to detect the circadian oscillation. The sub-circadian-cycle timescale dynamics in intron-based TFA could be due to noise in the data or could reflect meaningful

activity dynamics, which could inspire future investigations on the dynamic regulation of these TFs.

- For class 3 TFs showing circadian oscillation of intron-based TFA but not exon-based TFA (**Fig. S4B** bottom), it appears that the algorithm correctly discriminated between different dynamic patterns, but it is also challenging to speculate why intron-based TFA but not exon-based TFA was oscillating.

In the revised text, we have referred to these new plots and provided a brief discussion (**Line 363-368**).

- Fig. 4: How are the regulons for the shown TFs obtained? It is not stated here or in the methods. Also, is there any evidence that the levels/activity of these TFs change over time? While I can imagine that there be activity changes for the TFs downstream of T cell receptor signaling (e.g. NFAT, AP-1, and NFkB), it seems implausible that the activities of all these TFs could be changing within such a short time frame of 60 minutes. This result calls into question the process as to how TF regulons are defined.

We thank the referee for raising several important questions that should be addressed and clarified. In the following, we address each question separately.

- Regarding how TF regulons were obtained, the regulons were sourced from a carefully curated database named DoRothEA, which integrated multiple lines of evidence such as Chip-seq, gene expression correlation, and TF knockout studies. This information was described in various locations of the original manuscript, for example, in the method section under the subtitle 'The general pipeline for TFA estimation using RNA-seq data', and in the last paragraph of the introduction "by using existing RNA-seq data and literature-curated GRN [24]" (here, reference 24 is the paper that published the DoRothEA database). There are additional places throughout the original text that we referred to the DoRothEA database, including result and discussion sections. In the revised manuscript, we have edited the text to further clarify the source of TF regulons. Additionally, we have now provided a detailed analysis on how the choice of regulon affects the performance of TFA estimation (please refer to the response the referee 3's second comment).

- Regarding whether the detected global TF activity changes are due to potential issues with the TF regulon database, we think this is unlikely because of two reasons.
  - First, the regulon database DoRothEA has been widely used and cited in the community, and is regarded as one of the most reliable curated databases of mammalian gene regulatory network. And in our validation analyses (Fig. 2 and Fig. 3), we have shown that regulons from this database, together with intron-based method, can accurately capture TF activity dynamics (which were validated with data of a different modality).
  - Second, analysis in Fig. 4 captured the activity changes of well-known TCR

signaling TFs (such as those mentioned by the referee) within 60 min of TCR activation, which indicates that the regulons are likely reliable. Meanwhile, the large-scale changes of TF activities revealed biologically meaningful regulation, i.e., the two functionally distinct modules that change activities in the opposite direction over time. More specifically, one module enriched for homeostatic functions decreased its activity post TCR activation, while the other module enriched for stress responses increased its activity post activation. Critically, this biological insight cannot be obtained using exon-based method (please refer to the response to the last comment).

- Regarding the evidence for the presence of global TF activity changes within a short time post T cell activation, we agree that the observation of large-scale TF activity changes within a short time after TCR activation is indeed perplexing.
  - Because our assay uniquely analyzed the short-term transcriptional responses to TCR activation, we were not able to find relevant literature to backup our new finding that there is a large-scale TF activity change soon after TCR stimulation. Also, it is likely that such short-timescale changes in TF activity arise from post-translational regulation of the TFs, instead of transcriptional regulation, making it challenging to detect.
  - We thus resorted to our other newly collected dataset on the same cell line and under a similar condition, which measured the DNA accessibility changes within a short time after TCR activation using ATAC-seq and would allow us to address whether a regulon's promoter DNA accessibility also changed quickly after stress. We note that this dataset was collected for a different manuscript that we are currently preparing and is thus not to be published with this current manuscript.
  - By analyzing this ATAC-seq dataset, we found that DNA accessibilities around the promoter regions of genes in a regulon exhibited changes within 30 min post TCR activation for many TFs analyzed in Fig. 4, and such changes correlated with changes in the corresponding intron-based TF activities (**Fig. R1**, below). While this result is not a definitive proof that the activities of these TFs indeed change quickly post TCR activation, we feel that it provides support for our finding that the activities of many TFs can change within a relatively short time.

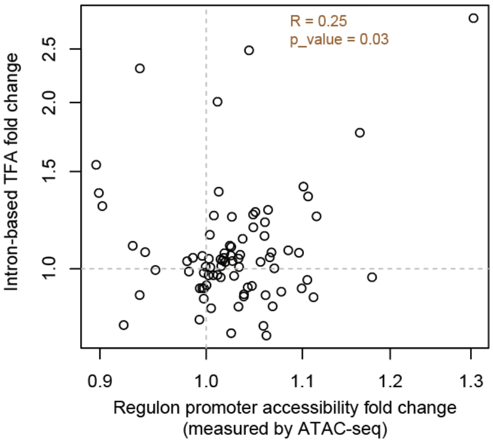


Fig. R1. Scatter plot showing the relationship between the fold-change of intron-based TF activity and the fold-change of regulon's promoter DNA accessibility. Fold-change of intron-based TFA was calculated using 0 and 60 min RNA-seq data in Fig. 4. Fold-change of promoter accessibility was calculated using 0 and 30 min ATAC-seq data (from our other manuscript). Pearson correlation and the associated p value were indicated (n = 81 TFs).

Together, we hope that these new analyses and clarifications have addressed the concerns raised by the referee. We have accordingly edited the text to point out that the observation of global TF activity changes necessitates further investigations (**Line 428-430**).

- Fig. 4: How do these TF modules obtained here compared to those obtained by analysis of exonic reads? If the authors are arguing that analysis of intronic reads better reveals these modules compared to analysis of exonic reads, they need to show the improvement to provide evidence that this technique is uniquely capable or at least more capable of generating biological insights.

We thank the referee for raising an important issue regarding biological insights. We would like to point out that a similar question was raised by referee one (i.e., the last comment by referee one) and was responded with additional analyses.

Briefly, in the original manuscript, we had already presented the comparison between intron-based TFA and exon-based TFA for TF modulation identification in the T cell dataset (please refer to Fig. 4 vs. Fig. S5A). In that comparison, we found that intron-based method (Fig. 4) uncovered two functionally opposing modules (i.e., one module enriched for homeostatic functions while the other module enriched for stress response functions) during T cell activation that change their activities in opposite ways, while exon-based method (Fig. S5A) yielded two overlapping functional modules (i.e., both enriched with related functions). We think these results address the concern raised here, i.e., intron-based method can indeed reveal unique biological insights compared to exon-based method.

In the revised manuscript, we have edited the related text to ensure that the biological insights from the T cell analyses are clearly explained and discussed (**Line 421-439**).

Reviewer #3: Using publicly available data, the authors presented evidence supporting the concept that transcription factor (TF) activities inferred from intronic reads (used as a proxy for pre-mRNA level) of the TF targeted genes can better recapitulate instantaneous TF activities compared to the exon-based reads in time-series RNA-seq data. More specifically, they showed that intron-based inferred NF-kappaB activities are better correlated with the measured NF-kappaB activities at nuclear localization level in a time-series single-cell RNA-seq data. Subsequently, they showed that intron-based TF activities improve the characterization of the temporal phasing of cycling TFs during circadian rhythm. They further applied the approach to their own data on transient T-cell responses to chemical stimulation and revealed two temporally opposing TF modules during T cell activation using Jurkat T cells. This paper proposed a simple and but effective analysis strategy to infer dynamic TF activities from time-series RNA-seq data. The idea is presented clearly in the manuscript. However, I have the following questions on specific technical aspects.

We thank the referee for the very helpful comments and suggestions. Based on these comments and suggestions, we have performed additional analyses to address the issues regarding regulon specificity, negative control regulon, among others. In the following we provide a detailed point-by-point response to the comments.

Major comments

1. In Fig. 2C, the authors compared VIPER, AUCell results to demonstrate the robustness of the proposed method. It was unclear how VIPER, and AUCell compute the correlation. Was it because the target genes were different? How is this related to robustness? Some explanation is necessary.

We thank the referee for pointing out that VIPER and AUCell methods were not clearly explained. To address this, we have now added additional sentences in the main text and the method section to explain how VIPER and AUCell were used to compute transcription factor activity. More specifically,

- In the main text we added two sentences to explain the overall procedure and refer the readers to the method section for more details on VIPER and AUCell **(Line 262-266)**.
- In the methods section, we added the following description to describe the two algorithms: AUCell is an algorithm to score the activity of each regulon in each cell. AUCell calculates the enrichment of the regulon as an area under the recovery curve (AUC) across the ranking of all genes in a particular cell, whereby genes are ranked by their expression value [22]. VIPER (virtual inference of protein activity by enriched regulon analysis) is an algorithm to estimate protein activity from gene expression data. From expression profile data, gene expression signatures (GES) are computed. Incorporating the regulon information, GES is then transformed into protein activity profile by aREA algorithm [23]. (**Line 611-618**)
- We note that the target genes (i.e., regulon) are the same for all algorithms used.

2. It is interesting to explore the relationship between the TF chromatin occupancy from the time-series TF ChIP-seq data and TF activities inferred from the time-series RNA-seq data. As the authors also mentioned in Discussion, the curated TF target genes are not cell-type specific. Thus, when examining the p53 activity, the authors could refine the target genes using the TF ChIP-seq data, i.e., focus on those target genes with associated ChIP-seq peak signals. I wonder what impact it would bring on the results in Fig.2.

We thank the referee for suggesting an analysis to examine the impact of regulon choice. We think this is a very necessary analysis, as it could demonstrate the importance of regulon choice and put forward a way to improve the accuracy of estimated TFA. In the following, we first present the result from the analysis, and discuss why the result has key implications.

As the referee pointed out, the DoRothEA database offers cell-type-non-specific regulons, which could limit the performance of TFA estimation in specific cell types. To address how regulon specificity may impact the analysis of p53 dataset, we first obtained a refined p53 regulon for MCF7 cells and performed the TFA analysis analogous to Fig. 2D-E. More specifically,

- We used p53 ChIP-seq data to select out potential p53 target genes, yielding 268 target

genes bound by p53 within TSS±2kb. We then intersected this gene list with the regulon from DoRothEA (195 targets), and obtained 42 target genes that are bound by p53 in MCF7 cells and appear in the DoRothEA regulon. We named these 42 genes as MCF7-specific p53 regulon.

- Using this refined, cell-type-specific regulon, we estimated p53 TFA and found that both intron-based and exon-based p53 activities display a sharp increase in the fold-change with respect to the unstimulated level (**Fig. S3D**). And in two replicates, both intron TFA and exon TFA exhibited a higher correlation with p53 DNA binding activity (**Fig. S3E**), indicating that TFA estimation is improved by using MCF7-specific p53 regulon.

These new results have important implications:

- First, while our current pipeline based on DoRothEA database offers a simple, effective approach estimate TFA, additional efforts such as curating a cell-type-specific regulon can increase the accuracy of estimated TFA. However, it should be noted that it is often challenging to obtain cell-type-specific DNA binding information.
- Second, the fact that our method can be improved by regulon refinement strongly supports the usefulness of the method, and we believe that future improvement in TF regulon database (i.e., GRN database) would help to boost the performance of our method.

We have now included these results in the revised manuscript and have thoroughly discussed the implications of these results (**Line 320-329**, **Line 486-488**), which we believe have greatly improved the manuscript.

3. The estimated TFA for a TF is dependent on the target gene set from a database and in this study, the DoRothEA database was used. Especially in the paper, the genes with confidence score A or B for a TF are selected for the TFA estimation. As a negative control, it would be good to know how different that would impact the TFAs if random target genes are selected.

We thank the referee for suggesting the inclusion of negative control in the analysis. We agree that it is important to show that the estimated TFAs are significantly different from the negative control. To address this, we focused on the p53 dataset and found that intron-based TFA is more significantly different from negative control compared to exon-based TFA. More specifically,

- We randomly chose negative control regulon (same size as p53 regulon) from 2995 genes (i.e., background targets in DoRothEA database), and 1000 sets of such random regulons were sampled. These random regulons were then used to compute intron-based and exon-based TFAs. By doing so, we found that compared to results of random regulons, intron-based TFA (computed using DoRothEA p53 regulon) displays a significantly higher correlation with p53 ChIP signal compared to the random controls (i.e., higher than 98.1% of the random regulons), and for exon-based TFA, the DoRothEA p53 regulon has a higher correlation than 99.1 % of the random regulons (**Fig. S3B**).

These new results have been included in the revised manuscript (**Line 309-310**, **Line 645-648**), which provides an important line of evidence for the validity of our method.

In addition, was mRNA-level of the TF itself considered? If a TF is expressed at a low level, do you still consider that the TF has a high activity level if the estimated TFA is a high value? Is there some conditioning on the TF mRNAs required? It was not clear from the paper.

We thank the referee for raising an important issue that needs to be clarified. The mRNA-level of the TF was considered in the pipeline. More specifically, in the 'RNA-seq experiment during Jurkat T cell activation' part, we mentioned that only TFs with non-zero expression values are considered. To improve clarify, we have now mentioned this in the figure legend of **Fig. 4** (**Line 921-922**).

Moreover, we would to point out that the mRNA expression level of a TF is generally not a good predictor of its activity, as we have shown in Fig. 2 for both NFkB and p53. And this is one of the key motivations for estimating TF activities using the expression of target genes.

Minor comments

1. The references sited seem to be inaccurate for several datasets used in the analysis.
Line2-4-Line 304: "We resorted to a public time-series RNA-seq data of circadian-entrained mouse livers collected with a relatively high temporal resolution (i.e., every 2 hours) [44] (Fig. 3A)." However, this paper does not have the mentioned datasets. They are from ref.43?

We thank the referee for pointing out the lack of clarity in dataset information. We note that the original citation was correct. The GEO accession provided by ref. 44 is GSE73554, and the download link for the specific dataset use is
https://ftp.ncbi.nlm.nih.gov/geo/series/GSE73nnn/GSE73554/suppl/GSE73554_WT_AL_Intron_Exon_RFP.txt.gz.

To improve clarity, we have now included detailed information of the datasets as supplementary table S1, which is mentioned in the Data Availability section (**Line 686-687**).

2. In the Fig.3 legend, class 1 and class I are used. Keep it consistent.

Thank you. We have now made the correction.

3. Provide GEO numbers to the public datasets used in the analysis.

Thank you. We have now included detailed information of the datasets as supplementary table S1, which is mentioned in the Data Availability section (**Line 686-687**).
